# Optical micromanipulation of nanoparticles and cells inside living zebrafish

**DOI:** 10.1038/ncomms10974

**Published:** 2016-03-21

**Authors:** Patrick Lie Johansen, Federico Fenaroli, Lasse Evensen, Gareth Griffiths, Gerbrand Koster

**Affiliations:** 1 Department of Biosciences, University of Oslo, Blindernveien 31, 0371 Oslo, Norway

## Abstract

Regulation of biological processes is often based on physical interactions between cells and their microenvironment. To unravel how and where interactions occur, micromanipulation methods can be used that offer high-precision control over the duration, position and magnitude of interactions. However, lacking an *in vivo* system, micromanipulation has generally been done with cells *in vitro*, which may not reflect the complex *in vivo* situation inside multicellular organisms. Here using optical tweezers we demonstrate micromanipulation throughout the transparent zebrafish embryo. We show that different cells, as well as injected nanoparticles and bacteria can be trapped and that adhesion properties and membrane deformation of endothelium and macrophages can be analysed. This non-invasive micromanipulation inside a whole-organism gives direct insights into cell interactions that are not accessible using existing approaches. Potential applications include screening of nanoparticle-cell interactions for cancer therapy or tissue invasion studies in cancer and infection biology.

## Abstract (web summary)

Manipulating the interactions of cells with their environment is usually done with cells *in vitro*, which does not reflect the complex *in vivo* system. Here the authors demonstrate micromanipulation of microparticles, bacteria and immune cells within live zebrafish using optical tweezers.

## Graphical abstract

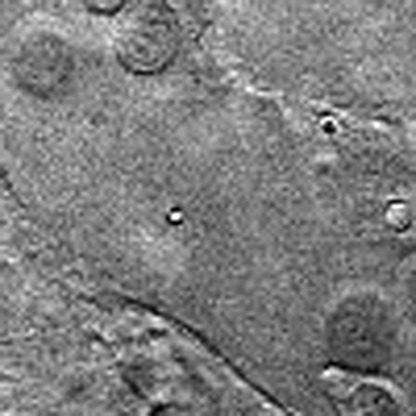

Many cellular mechanisms depend on regulation of the physical contact between biological structures. A prominent example of structures rich in cellular interactions are the blood vessels where the lumen is surrounded by a layer of endothelial cells; these form a crucial barrier between the blood vessel lumen and the surrounding tissue. This barrier is important for many functions, such as preventing blood clotting, in inflammation, in formation of new blood vessels and in the control of blood pressure^1^. Endothelial cells can interact with different immune cells in the blood, such as neutrophils and macrophages; they can also interact with cancer cells that can pass through the endothelium in the process of extravasation, as well as with blood-borne pathogens^1^.

As *in vivo* interactions are especially hard to characterize in detail since they are difficult to image and often occur at unpredictable positions and in short time windows, novel approaches are needed to increase the efficiency of ‘catching' these interactions and to study them in a controlled manner. In addition, to properly understand why and how cellular interactions are established, we need analytical methods operating at the sub-micrometre scale of cells and on the time scale of bond formation.

Optical tweezers (OT)^2^,^3^ are a highly sensitive and flexible micromanipulation tool that uses the force of light for non-invasive manipulation of nano- to micrometre sized particles. Moreover, by controlling the strength of the OT, interaction forces can be studied. Because of their versatility, OT have become a central tool for soft matter and life sciences in the last two decades^3^. Using this approach allows one to decipher when and where an interaction occurs, while parallel sensitive imaging and detection methods can be applied for detailed structural analysis. In order to achieve maximum control of the surrounding environment, and to simplify interpretation, a major part of OT experiments have been done *in vitro*, either in minimal reconstituted systems or with cells in culture. More recently, there have been developments towards optical trapping in the, technically more challenging, complex active environments inside living cells^4^,^5^,^6^,^7^. Ultimately, however, one would want to study interactions in a multicellular organism, where the interactions of interest occur deeper inside the organism, beneath layers of tissue. Towards this goal there has been one recent report describing optical trapping of erythrocytes in mouse^8^; however, these experiments were necessarily limited to red blood cells in thinner, superficial blood vessels in the ear.

Here we introduce the use of the zebrafish (ZF) larva for applying OT inside a living vertebrate. In the last years, the use of the ZF larva model has exploded in popularity for studying processes such as development and disease models^9^. An obvious advantage of using the ZF larva for OT is that it is optically transparent; moreover, many transgenic zebrafish lines are available with fluorescent cell types, such as macrophages and endothelial cells^10^. In a recent analysis of the biodistribution of different nanoparticles in the zebrafish we found that many of the nanoparticles bound tightly to the endothelium^11^. Here we take advantage of OT to follow and quantify these interactions. In another earlier project we have also followed the process of infection of *Mycobacterium marinum*, the organism of fish tuberculosis in the zebrafish and developed nanoparticle-based therapies against the disease^12^.

Using a flexible optical tweezers-imaging system in combination with zebrafish lines, we demonstrate here that many different types of structures, such as microinjected nanoparticles or bacteria, or different cell types such as macrophages (fluorescent) or erythrocytes can be identified and manipulated; this allows their interactions to be determined inside the living zebrafish. We analyse the details of particle–endothelium interactions and quantify ‘stealth' properties of particles; such stealth particles are designed to avoid interactions with phagocytic cells, important for using nanoparticles against cancer^13^. Using multiplexing, we show that a number of traps can be activated to ‘fish' out multiple nanoparticles from the blood stream simultaneously. We also demonstrate a procedure using OT where a region was first ‘cleaned' of erythrocytes after which the interaction details of nanoparticles with the endothelium could be studied in detail in a cell-free environment, thereby simplifying the interpretation. This experiment reveals the formation and detection of tethering nanotubes that are formed when adhered particles are pulled away from endothelial cells, providing convincing evidence that the particles were indeed tightly bound to the cell plasma membrane. Collectively, these data establish the zebrafish larva as a powerful model for optical trap micromanipulation and for analysis of *in vivo* interactions under controlled experimental conditions.

## Results and Discussion

### Optical trapping of nanoparticles inside living zebrafish

The optical transparency of the thin zebrafish larvae (Supplementary Movie 1; Supplementary Fig. 1) is unique among *in vivo* vertebrate models and one of the main reasons for its immense popularity as a model system. With this in mind, we tested whether the zebrafish was also ‘optically see-through' for the infrared laser beam of the optical tweezers and whether this approach could be used to manipulate different structures inside the living larva.

One recent emerging application of zebrafish as a model system is in the nanomedicine field^12^. For this, nanoparticles loaded with drugs and decorated on their surface with (potentially) targeting factors can be injected, enabling their biodistribution and blood circulation to be monitored using live imaging. To determine whether such injected particles can be trapped inside the complex environment of the fish, we injected latex particles into 2-day-old fish larvae (according to a recently developed protocol^12^, see also Methods, Supplementary Movie 2 and Supplementary Fig. 2). The particles readily distributed throughout the circulation of the blood stream and with time an increasing portion of the nanoparticles either adhered to the endothelium lining of the blood vessels or were taken up by macrophages.

**Figure 1:**
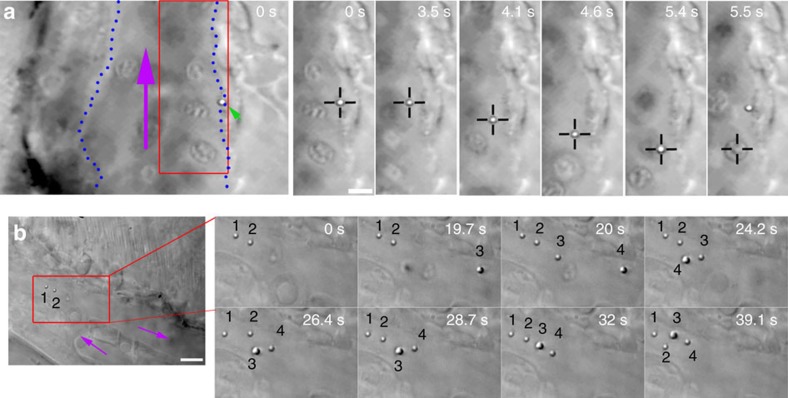
*In vivo* optical micromanipulation of microinjected particles. (**a**) A particle (green arrowhead) adhered to the endothelium of the caudal vein (indicated with blue dotted lines) is pulled away from the endothelium into the fast blood flow (purple arrow) using optical tweezers (black crosshairs). At time 5.5 s an erythrocyte is drawn into the trap. This replaces the particle in the trap which is subsequently pulled back towards the original adhesion point of the endothelium, presumably due to a connecting nanotube. Experiment repeated at least 80 times. (**b**) Four separate particles (numbered) are fished out of the blood flow and moved towards a sheltered region at the tip of the tail. Purple arrows indicate flow direction. Experiment was repeated at least 10 times. Scale bar, 5 μm.

After mounting the fish in the optical tweezers microscope (Supplementary Fig 3), we used transmission microscopy to select particles of interest that had adhered to the endothelium lining the caudal vein (Fig. 1a). Next, the OT were turned on and we were able to carefully move away some particles off the endothelium (Fig. 1a at 3.5 s, see also Supplementary Movie 3). Subsequently, the particles could be displaced and also moved against the direction of fast blood flow (200 μm s^−1^ in the vein and ∼700 μm s^−1^ in the artery^14^) indicating strong trapping. In the example shown (Fig. 1a), at time point 5.4 s an erythrocyte hits the optical trap, thereby dislodging the particle from the trap. Intriguingly, the dislodged particle was subsequently pulled back, spring-like, to the original adhesion point. This indicated that the particle was connected through a nanotube (as described previously^15^), which exerted a pulling force on the particle, that re-incorporated the tether into the endothelium after retraction. These manipulations could be done at a trapping laser power settings of 500 mW, about 10% of the maximum force available (corresponding to about max 75 mW in the sample). This demonstrates that statically adhering particles can be trapped in the zebrafish, making possible the investigating of details of interaction that are not possible with other methods.

After having established that an already adhered particle could be trapped, we next investigated whether it was possible to catch particles that were injected into the blood stream and moving with the flow at high speed^14^. To do this, we made use of the available time-sharing multiplexing option of the optical tweezers system; by scanning the trapping laser at high speed over several positions, multiple traps can be created that can be used to trap in parallel a high number of objects^16^. We thus distributed several ‘fishing' traps throughout the blood stream. In Fig. 1b (Supplementary Movie 4) using two traps, two particles (marked with ‘1' and ‘2') had already been moved towards the tail to a region with lower flow velocity outside the main blood flow (purple arrows). Next, another particle (‘3') was caught (19.7 s) and moved together with the two other particles while another particle (‘4') was trapped in the flow at *t*=20 s. To demonstrate the positioning control that the multiplexed tweezers allow *in vivo*, the particles were positioned first in a straight line (32 s) and next repositioned into a square shape (39.1 s). The trapping of multiple particles within the blood flow and the subsequent reorganization of their relative positions demonstrates the robustness of the trapping, and the versatility of experiments that are possible; for example, by establishing simultaneous contact between several particles and specific cells (Supplementary Movie 5; Supplementary Fig. 4).

Trapping was possible throughout the fish and up to depths of 100 μm away from the bottom cover glass (including zebrafish and medium, Supplementary Fig. 3) and we for example trapped cells and particles inside the beating heart. However, we found that most straightforward trapping could be done in the caudal vein and artery in the (thinner) tail region of the fish, at a depth of about 50 μm, where the arterial blood flow turns from retrograde transport towards the tail to anterograde transport towards the head of the fish. The experiments demonstrate that the adhesive properties of particles can be tested inside the living fish using micromanipulation, even in the fast flowing blood in the caudal artery. We successfully trapped and moved polystyrene particles of 200 nm to 1 μm diameter, but conducted most experiments with particles of 840 nm diameter (see Methods section, fluorescence emission peak at ∼700 nm) or 1 μm (non-fluorescent) because of their clear visibility and higher trapping stiffness^17^. Slowing down the blood flow using the anaesthetic tricaine^18^ facilitates optical trapping, and allows also smaller particles down to 200 nm to be moved, although this was more difficult due to the lower trapping stiffness and the fact that smaller particles were difficult to discriminate using bright field transmission microscopy. However, using confocal fluorescence microscopy the particles could be identified. The larger particles could be trapped without obvious heating damage for extended periods of time inside the vasculature with laser powers 100 mW to 3 W (about 15 mW to ∼450 mW in the sample). However, a few scattered darker areas (possibly pigments that had developed despite use of phenylthiourea (PTU), see Methods) interacted strongly with the OT, and when the OT was moved transiently over these areas they became visibly damaged, presumably due to heating. However, by avoiding these areas no discernable damage was observed over the course of the experiments.

### Trapping of cells in living zebrafish

Traditionally for experiments with optical tweezers spherical particles with high refractive index are used because they can be trapped with a high stiffness and they are especially suitable for trap calibration for quantitative experiments. However, for other applications, spatial micromanipulation is the crucial feature, as objects such as cells, particles or other nano–micron scale objects can be brought into contact with each other at well-defined time points and positions, opening up the possibility for analysing dynamics not accessible through passive observation.

**Figure 2:**
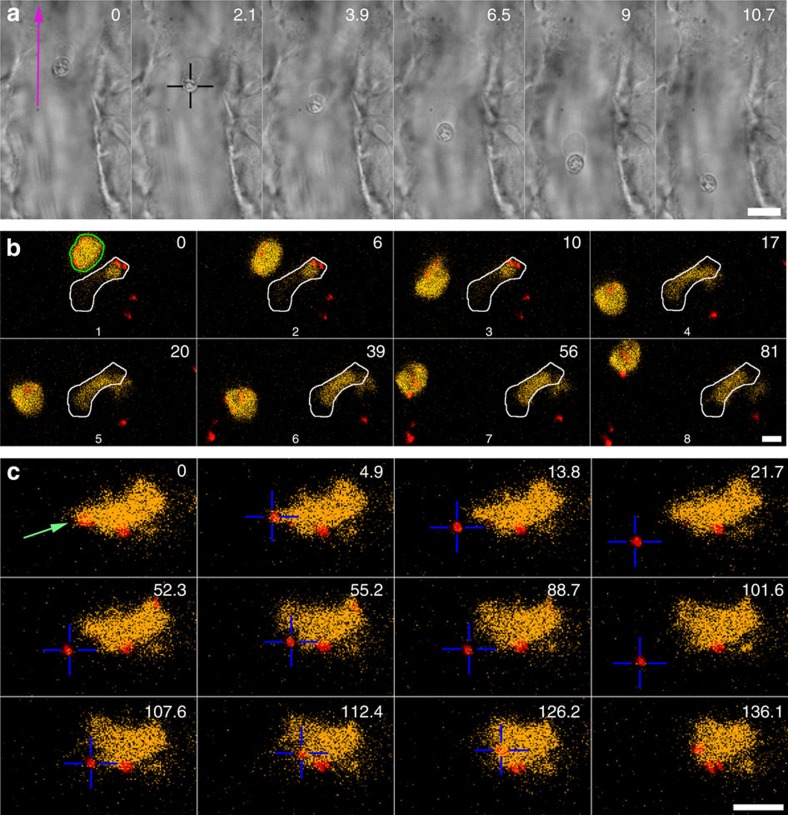
Trapping of erythrocytes and macrophages. (**a**) An erythrocyte is trapped and moved in the blood flow. Scale bar, 10 μm. Experiment repeated at least 10 times. (**b**) A blood-resident fluorescent macrophage (yellow, green outline in *t*=0 s) was micromanipulated and moved in 3D in a blood vessel. The white outline indicates another, non-mobile macrophage. The red dots are injected particles. Scale bar, 5 μm. Experiment repeated at least 5 times. (**c**) An injected particle (red colour) that was associated with a macrophage was tested for adhesion. First the particle was moved away (*t*=0–21.7 s) after which the OT was briefly shut off. This did not result in the particle flowing away with the blood, suggesting that a nanotube (not visible) was tethering the particle; next the particle was carefully brought into contact and moved away again (52.3–101.6 s), indicating that no strong binding was established. Finally, the particle was moved further into the macrophage with a higher pushing force after which the particle could not be detached anymore (*t*=107.6–136.1 s). Scale bar, 10 μm. Experiment was repeated at least 5 times.

As we found that particles can be easily and robustly trapped throughout the whole zebrafish, we next asked whether cells could also be micromanipulated inside the fish. In the absence of particles and using higher laser powers (about 2 W system setting, ∼250 mW in the sample), we activated the optical trap in the middle of the caudal vein, which almost instantly resulted in the immobilization of an erythrocyte in the trap. These cells could be stably held in position against the full flow of the blood stream (Fig. 2a; Supplementary Movie 6) and could even be moved in the direction against the blood flow. We found that the nucleus of the zebrafish erythrocytes^19^ was trapped most strongly. In Fig. 2a, a membrane in a ‘bag'-like shape can be observed behind the stably trapped nucleus, this deformation is caused by the drag force that the blood exerts and resembles the blebs that form due to local heating of cells^20^. This experiment shows that in the zebrafish optical trapping of erythrocytes is strong and seems more robust than that achieved in mouse ear^8^, where trapping had to be done gradually while making crucial use of the wall of the blood vessel. A possible explanation for this may be that erythrocytes in mice lack a nucleus.

Next, we tested whether other cell types could also be trapped in the zebrafish larva. However, it is not always trivial to identify different cell types based on solely using transmission imaging in the crowded and dynamic environment of a living organism. We therefore made use of a feature we implemented on the optical tweezers imaging system used here (adapted Nanotracker JPK Instruments AS, Berlin, see Methods), namely the possibility to do parallel trapping and confocal and transmission imaging (see also ref. 21). Using a transgenic zebrafish line with fluorescent macrophages, (Tg(mpeg1:mcherry))^22^ we thus set out to test the trapping potential of these cells. In this fish line, a relatively high number of macrophages are resident in the tissue and these cells cannot be manipulated using OT. However, several macrophages were also present freely in the lumen of blood vessels. In Fig. 2b (see also Supplementary Movie 7) an area with two macrophages in the caudal vein was selected; these macrophages could be identified using fluorescence imaging, one that was immobile (white contour) and another that could be moved using the OT. First, the macrophage was moved relative to the fish (using acousto-optic deflection-based repositioning; *t*=0–18 s and 39–81 s), while at *t*=20 s the whole stage, including the fish and the non-mobile macrophage, was moved (horizontally and axially), while the trap was held in a stable position. The combined trapping and confocal imaging in the zebrafish demonstrates that specific cell types can be identified and trapped using this versatile system. A plethora of transgenic zebrafish lines are available^10^ and different cell types and physiological questions can thereby be addressed. As another example, in Supplementary Movie 8 (and Supplementary Fig. 5) a particle (red colour, emission 640 nm) was moved in zebrafish with (green) fluorescent endothelial cells (Tg(fli1:EGFP)^23^.

We then asked whether particles that were associated with a macrophage could be moved using the tweezers; specifically, we wondered whether they were adhering to the macrophage surface or had been internalized by the cell. For this, we again used tricaine to slow the blood flow. In Fig. 2c (Supplementary Movie 9), a fluorescent macrophage was identified in the caudal vein of the zebrafish larva using confocal imaging. Next, a particle (red colour, indicated with a green arrow) was trapped with the tweezers and moved away, which resulted in its detachment from the macrophage (*t*=0–21.7 s), thus indicating that it was not inside the macrophage. Shutting the OT off briefly did not result in the particle flowing away with the blood as other particles (the red dots) can be observed to do while they move past the stationary macrophage and stationary red nanoparticles. This suggests that the particle remained tethered to the macrophage through a nanotube (not visible either because of it being in a different plane than the confocal imaging plane or because it was very thin and not very bright). Next, the particle was moved towards the macrophage again; however, no interaction could be observed (52.3–101.6 s). Finally, the particle was moved with a higher pushing force against the macrophage after which it adhered robustly, and it was not possible to detach the particle anymore (even at 5 W, the highest power available on our system). Macrophages play a central role in clearing of larger objects^24^, and it is crucial to understand this interaction for nanoparticle-based nanomedicine and delivery of drugs. The zebrafish-optical tweezers system makes it possible to study the dynamics of the interactions, make contact with a specific area of the cell, and may also be used to investigate the role of the contact forces with which particle interact when they are pushed against a macrophage in a controlled manner.

### Multiple traps for *in vivo* nanotube formation

**Figure 3:**
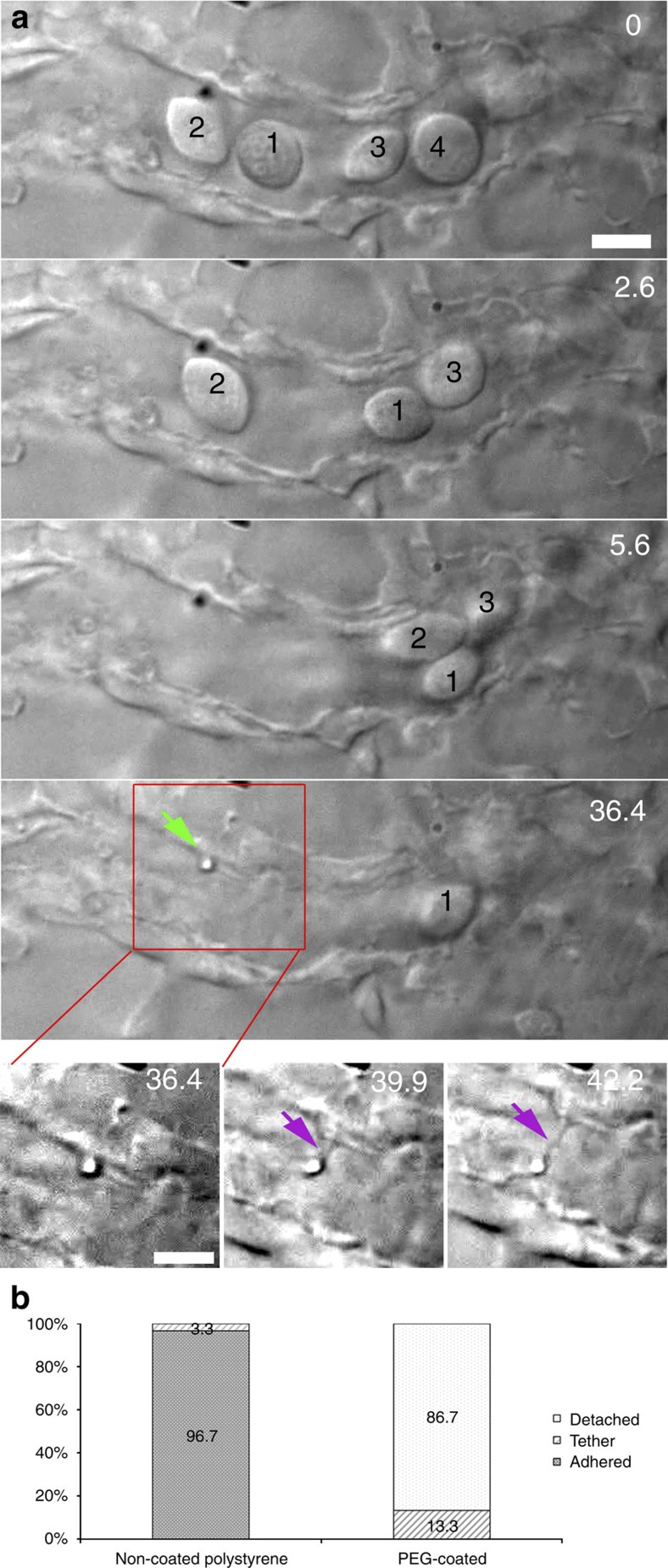
Multiple traps for *in vivo* nanotube formation in cell-free blood vessels (**a**) In a smaller blood vessel several erythrocytes are cleared (0–36.4 s) and fenced off, after which an adhered particle was moved away from the endothelium, and a tethering nanotube was formed (36.4–42.2 s). Scale bar, 5 μm. (**b**) Quantification of the adhesion probability of naked and PEG-coated particles, classified as: detachable (white), strongly adhered (solid) and tethered (lines). Experiment was repeated at least 60 times.

To study in more detail the particle adhesion to endothelial cells *in vivo*, a more complex system was tested involving multiple cells. In a zebrafish where the blood flow was slowed down (using tricaine), we first located a particle that adhered to the endothelium. Next, we applied several optical traps to clear the operation area of all cells: four erythrocytes that were present close to the adhered particle were removed (Fig. 3a; Supplementary Movie 10). First, cell ‘1' was moved away (behaving like a billiard ball pushing the other cells forward) and after moving it away it was kept trapped at a distance, allowing the assembly of a ‘fence' together with several other traps placed in adjacent positions. Next, cell ‘2' was moved and placed behind cell ‘1'; the ‘fence' formed by several optical traps prevented the cells from flowing and diffusing back into the operation region. In addition, cells that were out of focus experienced a scattering force that ‘blew' them away from the area of the fence. At *t*=36.4 s the earlier identified particle was next moved away from the endothelium. However, this particle did not fully detach, as a thin membrane protrusion was pulled out from the endothelial cell. Such membrane tethers or nanotubes have been studied in great detail *in vitro* since they can reveal information about, for example, membrane-cytoskeleton interactions, continuity of the membrane and its biophysical properties^25^,^26^,^27^. Our experiments using OT inside the live zebrafish larva show for the first time that nanotubes can be formed in a multicellular organism *in vivo* using active micromanipulation and that these nanotubes could function as a flexible adhesion, where adhered objects can be moved away while remaining tethered.

### Quantification of adhesion and ‘stealth' properties

Understanding how nanoparticles adhere to different cell types and how the dynamics of adhesion is regulated is crucial for nanoparticle-based drug delivery. We therefore wanted to test systematically the adhesion of particles to the endothelium cells lining the vessels. To do this we experimented with two types of particles: ‘naked'—and polyethylene glycol (PEG)-coated polystyrene particles (1 μm diameter). The PEG should provide a coat that lowers the non-specific affinity of the particles for cells such as macrophages. PEG has been widely used to provide ‘stealth' properties to nanoparticles designed for, for example, cancer therapy, facilitating a longer circulation time in the blood stream. In another project we used PEG-coated particles in ZF and effectively monitored their biodistribution and characterized their targeting and ‘stealth' properties to cancer cells^11^. Injected 1 μm particles adhering to the endothelium were detected and attempts were made to pull them away at a fixed laser power of 500 mW. For the naked polystyrene particles we found that the majority of the particles adhered strongly to the endothelium (Fig. 3b) and it was not possible to move them away at the laser power used (often also not at higher powers as they were strongly adhered). Out of 30 particles tested in the larvae, 29 could not be moved and only 1 could be pulled away but nevertheless remained connected through a tether and was pulled back after the trap was shut off (Fig. 3b, left bar). Next, we tested injected particles with a PEG-coating. These particles were also found to be adhered to the endothelium, and we determined the binding of these PEGylated particles (Fig. 3b, right). Out of the 30 PEG-coated particles that were found immobilized to the endothelium 26 could be detached, whereas four formed a tether. None of these particles were attached so strongly that it was impossible to move them away from the endothelium. This demonstrates that PEG lowers the binding affinity of the polystyrene particles for endothelial cells *in vivo*, even though they still adhered to some extent to the endothelium.

The fact that the PEG-coated particles more often form tethers indicates that the particles do adhere, but with a significantly lower strength than non-coated particles. The initial force required for tether formation depends strongly on the size of the adhesion zone and more frequent tether formation implies that the area of adhesion to endothelial cells is smaller in the PEGylated-particle-case. Naked-particles likely adhere through larger areas resulting in a higher threshold force for nanotube formation^15^,^28^ than the tweezers can provide.

We then investigated whether heparin^29^ would affect the binding of nanoparticles to the endothelial lining, given that proteins and drugs with heparin binding sites that bind the endothelium can be detached in the presence of heparin. We evaluated this first by comparing the circulation of fluorescent particles in ZF injected with mixtures (3 nl) of particles (non-coated polystyrene) together with or without heparin (concentrations 40 and 100 mg ml^−1^). Confocal fluorescence imaging revealed no difference in adhesion properties in the presence or absence of heparin even at the highest drug concentration. We then used optical tweezers to evaluate whether the adhesion properties of particles had changed on a ‘more subtle level' in the presence of heparin. As with the PEG-coated particles (Fig. 3b) we used the OT to pull on nanoparticles adhered to the endothelium (500 mW laser power). These experiments show that there was no significant difference in the adhesion properties in the presence or absence of heparin (Supplementary Fig. 6).

### Optical trapping and manipulation of injected bacteria

After having demonstrated trapping of injected particles and naturally occurring zebrafish cells inside the living zebrafish larva, we tested whether this approach can also be applied to study injected bacteria. Bacteria were amongst the first biological particles to be optically trapped *in vitro* in aqueous solutions^2^, but as far as we know there are no reports of trapping bacteria inside living vertebrates. Rod-shaped bacteria are more difficult to trap than spherical (polystyrene) particles and they are smaller than macrophages and erythrocytes. As a proof of principle of optical manipulation of bacteria in zebrafish, we injected the fish bacterium *M. marinum*, which causes fish tuberculosis, and which has been widely and effectively used as a model for human tuberculosis in the zebrafish larva^12^,^30^.

**Figure 4:**
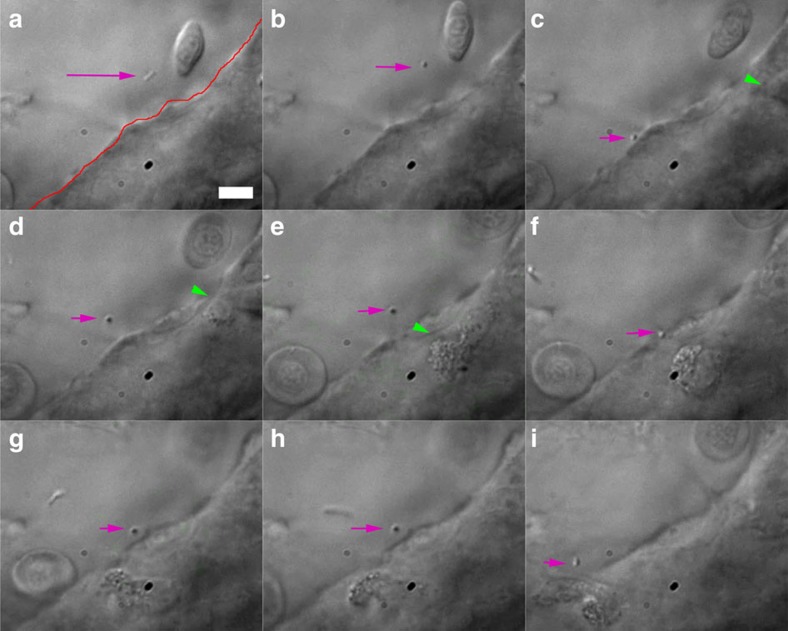
Trapping of injected bacterium. (**a**) A diffusing bacterium (purple arrow) is (**b**–**d**) trapped, pushed against and moved away from the endothelium (red line in **a**). This seems to activate an immune cell (green arrowheads), which moves towards the contact point (**c**–**f**). Repeated contact with the endothelium seems to again attract the attention of the crawling cell, which (**g**–**i**) finally moves into the vein. The total duration of experiment is ∼2 min. Scale bar, 10 μm. Experiment was repeated at least 4 times.

For this experiment we selected a region where the blood flow was very slow (again using tricaine), and using either fluorescence or transmission imaging, we could detect the presence of (red fluorescent) bacteria that had been injected (Fig. 4). Next, a bacterium was trapped and moved against the endothelium using the OT (Supplementary Movie 11). Before the trap was activated, the bacterium was seen moving in a Brownian manner through the blood vessel, rotating and diffusing. It could be imaged in a snapshot while oriented in the imaging plane (Fig. 4a, see also Supplementary Movie 11). Activating the optical tweezers (Fig. 4b) resulted in the bacterium becoming orientated in a direction perpendicular to the imaging plane (in the direction of the tweezer beam). This reorientation is expected for elongated objects. Next, the bacterium was pushed against the endothelium (Fig. 4c). Intriguingly, in Fig. 4d a cell (presumably a macrophage^31^) could be observed crawling towards the contact point. After the bacterium was repeatedly moved against the endothelium, the immune cell was seen to arrest its movement and finally seemed to ‘decide' to move across the endothelial barrier (Fig. 4g-i). Macrophages are known to collect bacteria^31^, and controlling the position and timing of bacteria and interactions with different cells makes it possible to study this phenomenon in a controlled manner. The OT can for example be used to determine how long a bacterium needs to stay in contact to adhere to a cell or to evoke a response, or whether multiple bacteria will increase the recruitment of macrophages or influence phagocytosis dynamics.

We also tested the effects of anti-inflammatory drugs on the migration of immune cells to bacteria-invaded areas. Our preliminary experiments using fish lines with either fluorescent neutrophils or fluorescent macrophages confirmed that the anti-inflammatory drugs diclofenac and indomethacin^32^ inhibited the recruitment of neutrophils (and macrophages to a lesser degree, unpublished results) to sites where bacteria had been injected^12^. However, these experiments need a systematic follow-up for their significance to be verified.

Collectively, the experiments we have described demonstrate for the first time active micromanipulation of a full scale of nano- to micron-sized structures inside a living vertebrate using the transparent zebrafish larva. The manipulated structures ranged from injected nanoparticles and bacteria to naturally occurring zebrafish cells as erythrocytes and macrophages.

We foresee many uses of this approach such as (but not limited to), the characterization of interaction properties of nanoparticles with specific cells for nanomedicine applications. In particular the properties of nanoparticles could be studied, for example, by functionalizing them with ligands for targeting to specific cells or with coats such as PEG to prevent interactions with other cells. Alternatively, optically manipulated nanoparticles releasing a specific compound could be brought in the proximity of the organismal structure of interest for testing of local cellular responses to chemicals (as has been already demonstrated elegantly with cells *in vitro*^33^) such as for studies of vascular function and endothelial integrity.

Controlled investigations of recruitment and activation of immune cells, by micromanipulating bacteria, other microorganisms or antigen-coated particles to specific regions in the organism will make possible to investigate adhesion to and activation/recruitment of immune cells, for example in the presence of anti-inflammatory drugs, especially in combination with imaging. Finally, quantitative optical tweezers have been instrumental to understanding cellular biomechanical properties and their regulatory role in function. However, this has been mostly done *in vitro* with cells in culture, and the work presented here opens up many possibilities to perform such experiments throughout a living vertebrate.

## Methods

### Zebrafish care and treatment

Two lines of transgenic ZF larvae were used, Tg(fli1:EGFP) and Tg(mpeg1:mcherry) with green fluorescent endothelial cells and red fluorescent macrophages, respectively. The ZF larvae were kept in Petri dishes containing salt-containing water^18^ with 0.003% w/v phenylthiourea (PTU, Sigma-Aldrich, St. Louis, USA) to keep the fish transparent by preventing pigmentation in retinal epithelium and melanophores^34^. All experiments were done at 28.5 °C. Experiments were conducted in agreement with the ethical provisions enforced by the Norwegian national animal research authority (NARA).

### Microinjections of zebrafish larvae

Injections were done using a glass micropipette (Harvard apparatus, Holliston, USA), with an outer diameter of 1.0 mm and inner diameter of 0.78 mm. The glass micropipettes were made using a micropipette puller Model P-97 (Sutter Instruments Co., Novato, USA). Manipulation of the micropipette was done using a Narishige MN-153 micromanipulator (Narishige, London, UK), and injection time and pressure was controlled using a FemtoJet Express micro injector (Eppendorf, Hamburg Germany). Visualization of the ZF larvae during injections was done with a stereomicroscope (Leica DFC365FX with a × 1.0 Planapo lens).

For all injections, the ZF larvae were anaesthetized using (0.5–2 mg ml^−1^) tricaine (Finquel, Argent Laboratories, Redmond, USA) in appropriate solution^18^. The ZF embryo was then placed on a gel made of hardened 2% agarose (Sigma-Aldrich) in water, and excess fluid was removed from around the ZF embryo using a pipette. This was done to immobilize the fish before injections.

Nanoparticles of five different sizes were used; four Fluoresbrite Microparticles (Polysciences Inc., Warrington, USA) with diameters of 100, 200, 500 and 1000, nm, and SPHERO particles (Spherotech Inc., Lake Forest, USA) with 840 nm diameter. The 200 nm Fluoresbrite particles contained fluorescein dye (yellow–green) with excitation maximum at 441 nm, while the 500 nm and 1 μm nanoparticles contained coumarin dye (bright blue) with excitation maxima 360 nm. The 840 nm SPHERO nanoparticles (Spherotech Inc.) contained sky blue, with excitation at 640 nm. For the experiments on the ‘stealth' effect of PEG coating, the Fluoresbrite nanoparticles were modified with MPEG5000-NH2 on the surface^11^. The nanoparticles were diluted in PBS to a concentration of 2 × 10^8^ nanoparticles per ml and loaded into a glass micropipette. Subsequently, 3–6 nl was injected into the posterior cardinal vein of the ZF larvae. *M. marinum* carrying the fluorescent reporter construct DsRed was injected (250 c.f.u.) in the posterior cardinal vein at 48 h post fertilization, as in ref. 12.

### Sample preparation for optical tweezers experiments

Following injection, the ZF larvae were moved to a Petri dish containing a tricaine solution. The concentration of tricaine was between 0.1 and 0.4 mg ml^−1^ in salt water^18^ (depending on how slow blood flow was desired for the experiment).

Two parallel lines of silicone grease were applied to a 22 × 60 mm cover glass using a hypodermic needle and a syringe. The length of the grease lines was ∼30 mm in length and the distance between the two lines was ∼15 mm. Between these silicone lines the ZF embryo was placed in ∼100 μl tricaine solution using a pipette. Using a small paint brush or hair loop, the embryo can be manipulated into a suitable position so that its body and tail are close to the cover glass (Supplementary Fig. 3, right), which facilitates optical trapping. A 22 × 22 mm coverslip (Karl Roth nr 1, thickness 0.13–0.16 μm) was carefully placed on top, resting on the two lines of silicone. The coverslip was pushed down onto the silicone, carefully, in order not to damage the embryo. It is important that the 22 × 22 mm coverslip is pushed far enough down to make sure the ZF embryo does not float around or move during the experiment. Excess fluid was removed from the edges using filter paper, or fill up the remaining space between the coverslips was filled with an embryo water-tricaine solution if necessary. The remaining openings between the cover glasses were sealed using clear nail polish. The sample was next mounted (with the 22 × 22 mm cover glass downwards) on the sample stage in the optical tweezers microscope.

### Optical tweezers and imaging microscope

An adapted version of the NanoTracker2 system (JPK Instruments AG, Berlin) was used. This custom-built system was developed in collaboration with JPK Instruments for parallel confocal, transmission and optical trapping (Supplementary Fig. 3, left). A 1,064-nm trapping laser (5 W) was split into two polarizations for independent trapping. One of these beams is controlled through a piezo-mirror and the other passes through Acousto-Optic Deflectors for position control and multiplexing. The trap position relative to the fish could also be controlled through movement of the whole sample with a piezo stage.

The optical trapping system was merged on a NIKON C2 confocal microscope with a × 60 (numerical aperture (NA) 1.2, WD 0.27 mm) water immersion objective for imaging and trapping, and we used Zeiss' ‘Immersol' immersion fluid for water objectives (*n*=1.334). Transmission light was focused in the sample through a × 60 water dipping condenser (NA 1, WD 2.5 mm). To be able to image in parallel in confocal mode, the ∼700 to 900 nm band was used for transmission, which does not interfere with the confocal imaging.

### Quantification of adhesion with and without PEG coating

For the quantification of the binding affinities of the 1 μm polystyrene nanoparticles with and without PEG, we used three individual fishes for both PEG- and non-coated nanoparticles. In each of these 6 individuals 10 particles were trapped and manipulated using 500-mW laser power. Three possible outcomes were considered in the experiment: (1) the particle remained adhered to the endothelium; (2) the nanoparticle detached from the endothelial cell; or (3) a tether was pulled from the cell, allowing the particle to be moved away but maintaining the connection to the endothelium.

## Additional information

**How to cite this article:** Johansen, P. L. *et al.* Optical micromanipulation of nanoparticles and cells inside living zebrafish. *Nat. Commun.* 7:10974 doi: 10.1038/ncomms10974 (2016).

## Supplementary Material

Supplementary Information Supplementary Figures 1-6

Supplementary Movie 1 Transparent zebrafish. The transparent zebrafish larva can be used for whole organism live imaging.

Supplementary Movie 2 Injection of red nanoparticles into zebrafish with green endothelium blood vessels.

Supplementary Movie 3 Trapping of adhered particle. A particle is pulled away from the endothelium in the caudal vein and moved into the blood flow. Experiment repeated at least 80 times. Movie of (rotated) still images in Figure 1a.

Supplementary Movie 4 Simultaneous trapping of multiple particles in the tail region. Movie of Figure 1b. 4x real time. Experiment repeated at least 10 times. Vibration is due to the fish heart pumping.

Supplementary Movie 5 Trapping of two particles. Two particles are trapped inside the blood flow and moved against the endothelium. Experiment repeated at least 10 times.

Supplementary Movie 6 Trapping of an erythrocyte in blood flow. Movie of Figure 2a. Sped up 4 times. Movie is rotated 90 degrees relative to the Figure. Experiment repeated at least 10 times.

Supplementary Movie 7 Trapping and moving a fluorescent macrophage in the vein. Movie of Figure 2b. Total movie length is 1 min 39 s. Experiment repeated at least 5 times.

Supplementary Movie 8 Displacing and feeding a particle to a macrophage. A (far red) particle is moved repeatedly against a fluorescent macrophage. Movie of Figure 2c. Experiment repeated at least 5 times.

Supplementary Movie 9 Trapping of particle (red, fluorescent) in fish with green fluorescent endothelium.

Supplementary Movie 10 Multiple traps for in vivo nanotube formation in cell-free blood vessels. After clearing erythrocytes from a vein, an adhered particle is moved away and a tethering nanotube can be identified. Movie of Figure 3a. 8x real time. Experiment repeated at least 60 times.

Supplementary Movie 11 In vivo trapping of bacterium. Movie of Figure 4. A bacterium (M. marinum) is trapped and moved against the endothelium which recruits an immune cell. Movie 2x real time. There are 3 (~2 second) jumps when the video recording was restarted. Experiment repeated at least 5 times.
