# Supplementary material for: Optical micromanipulation of nanoparticles and cells inside living zebrafish
Source: Nat Commun. 2016 Mar 21;7:10974. doi: 10.1038/ncomms10974 (PMC4802177; doi:10.1038/ncomms10974)
Supplement: Supplementary Information — Supplementary Figures 1-6 [file ncomms10974-s1.pdf]

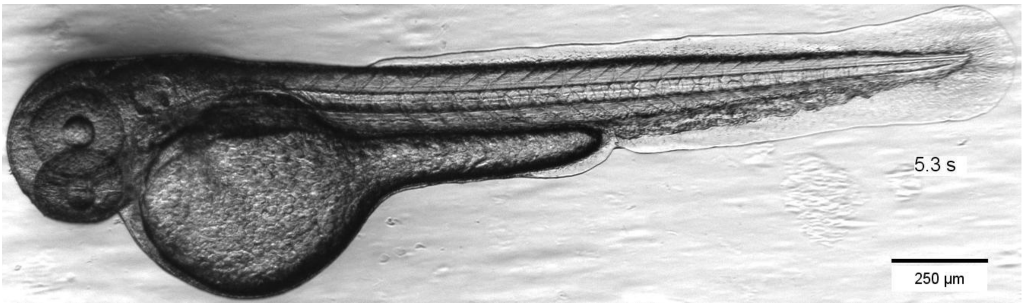

- **Supplementary Figure 1: Transmission bright field image of zebrafish larva.**

  Snapshot of Supplementary Movie 1.

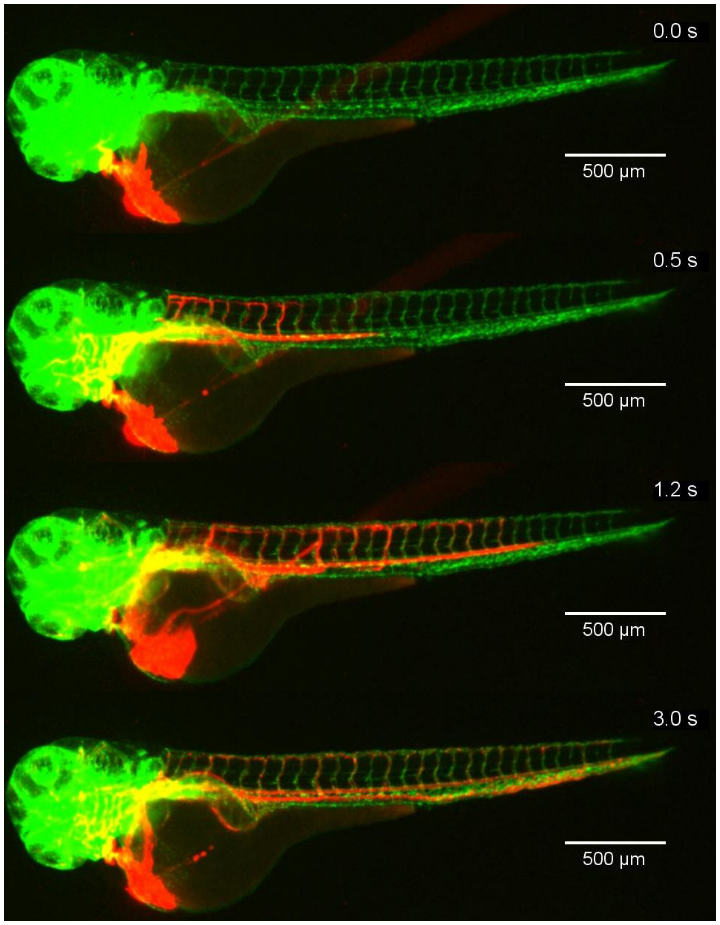

- **Supplementary Figure 2: Injection of nanoparticles into the zebrafish with blood vessels with green fluorescent protein labeled endothelium.** Snapshots of Supplementary Movie 2. At t=0 microinjection was started. The particles (fluorescent, red) distribute throughout the zebrafish within 3 seconds.

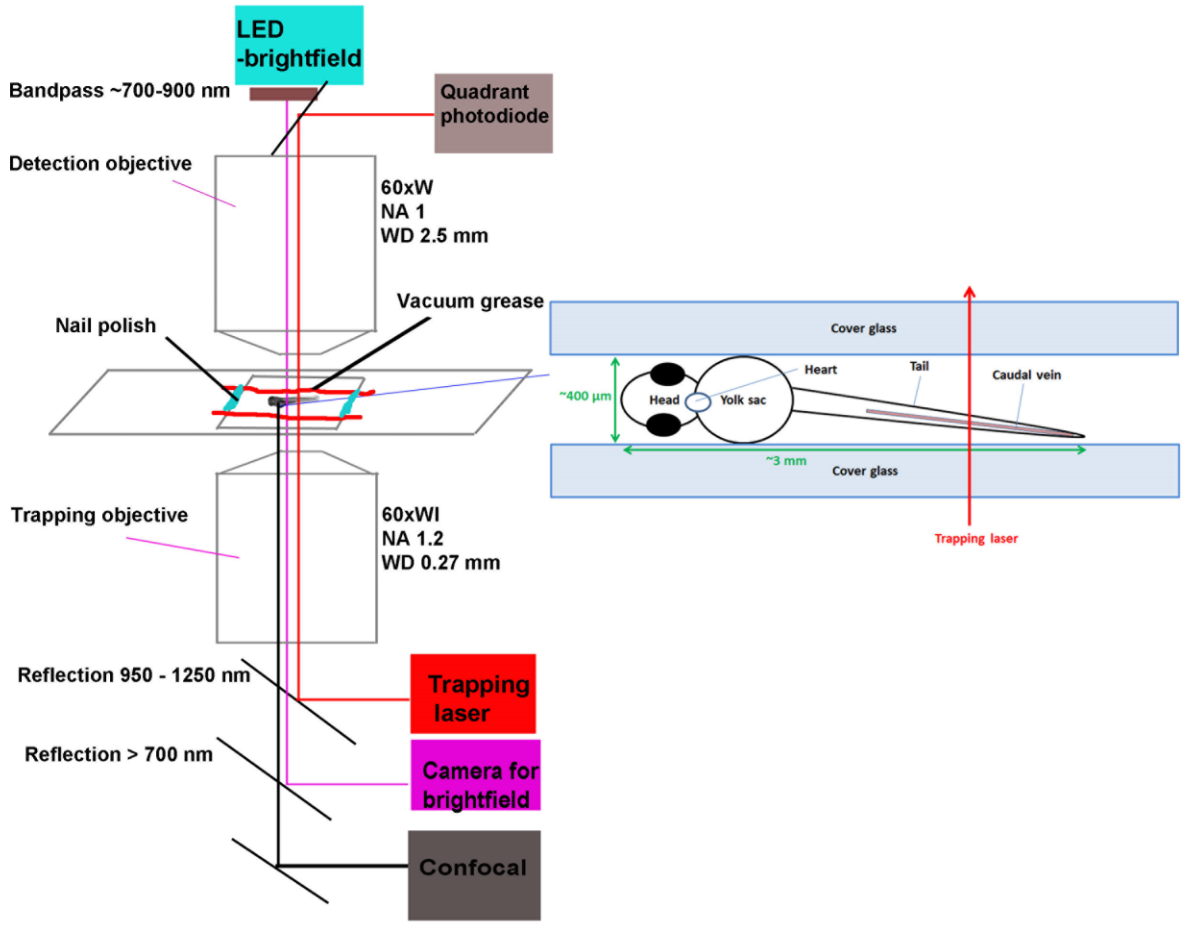

- **Supplementary Figure 3: Sketch of experimental set-up (left side).** The zebrafish larva is mounted in the optical tweezers microscope between two cover glasses. A 60x water immersion objective is used for both imaging and focusing of the optical trap. Transmission light is focused in the sample using a 60x water dipping objective, which is also used for detection on quadrant photodiodes. For parallel trapping, confocal and transmission imaging, the wavelength band between 700 and 900 nm is used for transmission. **(right side)** Schematic drawing of the fish (side-view) is given which indicates the position of the different relevant regions.

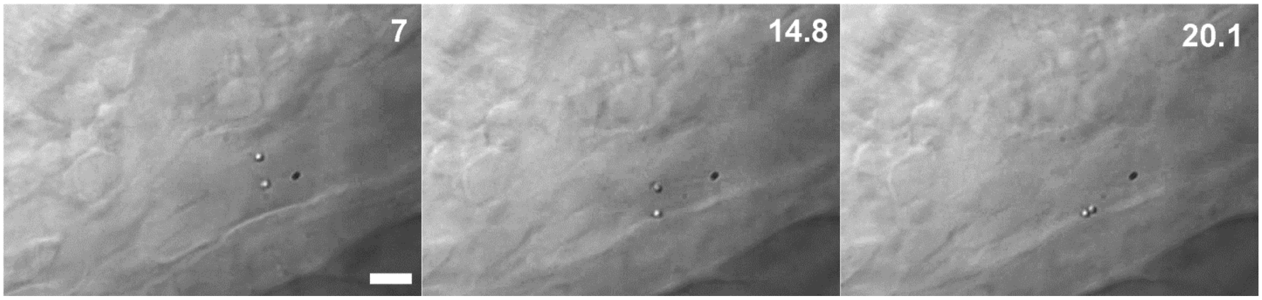

- **Supplementary Figure 4: Two particles are trapped from the blood in the tail region of the fish and moved towards the endothelium simultaneously.** Snapshots of Supplementary Movie 5. Experiment repeated at least 10 times. Time in seconds. Bar is 10 µm

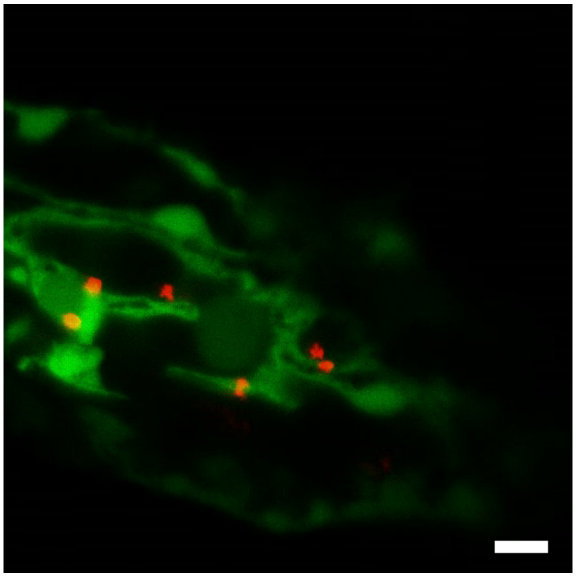

- **Supplementary Figure 5: Trapping of fluorescent (red) particle in zebrafish with fluorescent (green) endothelium.** Snapshot of Supplementary Movie 8. Experiment repeated at least 10 times. Bar 10 µm.

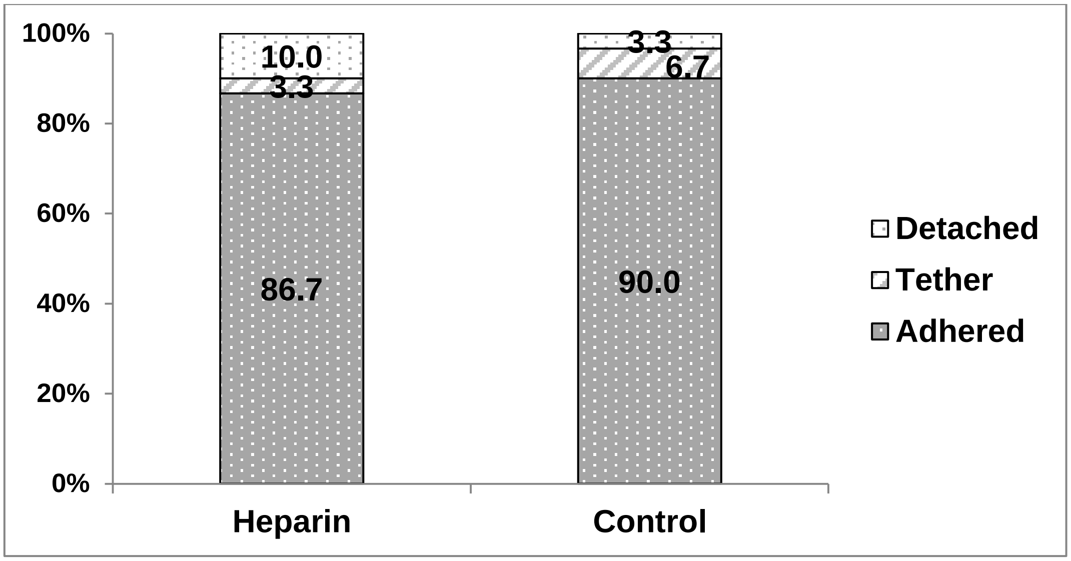

- **Supplementary Figure 6:** Quantification of adhesion properties in the presence of heparin.
